# 

**Published:** 1901-03-09

**Authors:** 


### [illegible]ICE TO CORRESPONDENTS.

[illegible]., Letters, Books for Review, and other matters intended for [illegible]or should be addressed The Editor, "The Hospital," The [illegible]al Building, 28 & 29 Southampton Street, Strand, W.C. [illegible] Editor cannot undertake to return rejected MS., even when [illegible]mpanied by stamped directed envelope.

**Notice to Advertisers and Subscribers.**

All Advertisements, Orders for Copies of The Hospital, and Business Communications should be addressed to **THE MANAGER (Not to the Editor),** The Hospital, 28 & 29 Southampton Street, Strand, London, W.C.

The Cost of Subscription, post free, is as follows:—Per week, 2½d.; per quarter, 2s. 8d.; per half-year, 5s. 3d.; per annum, 10s. 6d. Foreign:—Per annum, 15s. 2d., payable, in all cases, in advance.

**NOTICE.—Vol. XXVIII. of "The Hospital" (April to September, 1900), in handsome cloth binding, is now on sale at the Office of this Journal, price, 7s. 6d. Cases for binding the Numbers contained in this Volume 1s. 6d. Index to Vol. XXVIII., 2½d., post free.**

**The Monthly Part for March is now ready. Price 6d., by post 8d.**

### The Student of Medicine.

### APPOINTMENTS.

**Comyns Berkeley,** B.A., M.B., B.C., appointed Obstetric Registrar to Middlesex Hospital. **J. Buist,** M.B.Lond., appointed Surgeon to the A Division of the Cardiff Police. **Arthur Bulleid,** L.R.C.P., L.R.C.S.Edin., appointed Medical Officer of Health to the Midsomer Norton Urban District Council. **John W. Byers,** M.D., appointed Examiner in Midwifery and Gynæcology to the Royal University of Ireland. **C. I. Ellis,** M.B., C.M.Aberd. appointed Medical Officer and Public Vaccinator for the No. 3 District of Battersea of the Wandsworth Union. **Thos. Evans Flitcroft,** L.R.C.P.Edin., L.F.P.S.G., appointed Honorary Surgeon to the Bolton Infirmary. **M. B. Hay,** M.R.C.S., L.R.C.P.Lond., appointed House-Surgeon at the North Devon Infirmary, Barnstable. **M. Pittard,** M.R.C.S.Eng., L.S.A., appointed Surgeon to the B Division of the Cardiff Police. **S. H. Richards,** M.B., C.M.Edin., appointed District Medical Officer of the Middlesbrough Union. **Alexander Robb,** M.B., C.M.Edin., appointed Medical Officer of Health for the Burgh of Paisley. **G. Melmoth Scott,** M.D., B.C.Cantab., appointed Senior House-Surgeon to the Birmingham and Midland Eye Hospital. **M. E. Smallwood,** M.R.C.S., L.R.C.P.Lond., appointed Second Assistant Medical Officer to the Shoreditch Parish Infirmary. **J. L. Treharne,** M.R.C.S.Eng., L.S.A., appointed Chief Surgeon and Surgeon to the C Division of the Cardiff Police. **J. W. Thomson Walker,** M.B., F.R.C.S., appointed Honorary Pathologist and Bacteriologist to the Paddington Green Children's Hospital.

St. Thomas's Hospital.—The following gentlemen have been selected as House Officers :– House Physicians: **Z. Mennell,** L.R.C.P., M.R.C.S. ; **F. B. Skerrett,** B.Sc.Lond., L.R.C.P., M.R.C.S. ; **C. F. Selous,** L.R C.P., M.R.C.S. (*Extension*) ; **H. H. R. Clarke,** L.R.C.P., M.R.C.S. (*Extension*). Assistant House Physicians: **J. E. H. Sawyer,** B.A., M.B., B.Ch.Oxon ; **J. L. Lock,** B.A., M.B., B.C.Cantab., L.R.C.P., M.R.C.S. House Surgeons: **T. H. Edwards,** L.R.C.P., M.R.C.S. ; **B. S. Wills,** L.R.C.P., M.R.C.S. ; **T. S. Taylor,** M.A., M.B., B.C. Cantab. ; **T. Burfield,** M.A., M.B., B.C.Cantab., L.R.C.P., M.R.C.S. Assistant House Surgeons: **C. A. R. Nitch,** M.B.Lond., L.R.C.P., M.R.C.S. ; **W. H. O. Woods,** M.B., B.C.Cantab. ; **S. Hunt,** L.R.C.P., M.R.C.S. ; **A. S. Grimwade,** L.R.C.P., M.R.C.S. Obstetric House Physicians: (*Senior*) **E. W. Hedley,** M.A., M.B., B.C.Cantab. ; (*Junior*) **A. E. Martin,** M.A., M.B., B.C.Cantab., L.R.C.P., M.R.C.S. ; Ophthalmic House Surgeons: (*Senior*) **J. F. Cunningham,** L.R.C.P., M.R.C.S. ; (*Junior*) **H. S. Harris,** L.R.C.P., M.R.C.S. Clinical Assistants in the Special Department for Diseases of the Throat: **T. W. S. Paterson,** M.A., M.B., B.C.Cantab (*Extension*) ; **H. S. Stannus,** L.R.C.P., M.R.C.S. Clinical Assistants in the Special Department for Diseases of the Skin: **W. C. Mence,** L.R.C P., M.R.C.S. Clinical Assistants in the Special Department for Diseases of the Ear: **L. H. C. Birkbeck,** B.A., M.B., B.Ch.Oxon (*Extension*) ; **T. W. S. Paterson,** M.A., M.B., B.C.Cantab. (*Extension*). Clinical Assistants in the Electrical Department: **L. S. Dudgeon,** L.R.C.P., M.R.C.S. (*Extension*) ; **A. Bevan,** L.R.C.P., M.R.C.S.

